# An eye-tracking and visual analogue scale attractiveness evaluation of black space between the maxillary central incisors

**DOI:** 10.1590/2177-6709.26.1.e211928.oar

**Published:** 2021-03-22

**Authors:** Ahmad AL-LAHHAM, Paulo Henrique Couto SOUZA, Caio Seiti MIYOSHI, Sérgio Aparecido IGNÁCIO, Thiago Martins MEIRA, Orlando Motohiro TANAKA

**Affiliations:** 1 Pontifícia Universidade Católica do Paraná, Escola de Ciências da Vida, Programa de Pós-Graduação em Odontologia, Área de Concentração em Clínica Odontológica Integrada - Ortodontia (Curitiba/PR, Brazil).; 2 Pontifícia Universidade Católica do Paraná, Escola de Ciências da Vida, Programa de Pós-Graduação em Odontologia, Área de Concentração em Clínica Odontológica Integrada - Estomatologia (Curitiba/PR, Brazil).; 3 Pontifícia Universidade Católica do Paraná, Escola de Ciências da Vida, Disciplina de Bioestatística (Curitiba/PR, Brazil).; 4 Universidade do Estado da Bahia, Departamento de Educação XII (Guanambi/BA, Brazil).

**Keywords:** Black space, Perception, Esthetics, Eye-tracking

## Abstract

**Objective:**

To study the influence of black space between the maxillary central incisors on the aesthetic visual perception of the face, via eye-tracking and visual analogue scale (VAS).

**Methods:**

Black space between the central incisors was created, for both sexes, as follows: control, 1-mm black space, 2-mm black space and 3-mm black space. Ninety raters participated in this study, divided into three groups: 30 laypeople, 30 nonorthodontists, and 30 orthodontists. After the visual calibration of each observer, eight photographs were presented in the Ogama^®^ software concomitant with the use of the hardware The Eye Tribe^®^. Ogama generated information depending on the eye-tracking of each rater, regarding the time until the first fixation, time of fixation, heatmap, scanpath, and total time of fixation, to evaluate the areas deemed to be of interest according to the raters. Later on, the VAS was used, where each rater evaluated the images in an album on a scale of zero to 10 points.

**Results:**

The eyes and mouth were the areas more often noticed by the raters according to the heatmaps, while no significant difference was observed in time until the first fixation between the three groups of raters (*p*> 0.05). However, regarding the time of fixation on the mouth, a significant difference was observed (*p*< 0.05) when comparing the three groups.

**Conclusion:**

Black space has a negative effect on the aesthetic perception of the face. The amount of attention on the mouth is correspondent to the size of the black space.

## RESUMO

**Objetivo:**

Avaliar a percepção visual e estética do espaço negro entre os incisivos centrais superiores, via rastreamento do olhar e escala visual analógica (EVA).

**Métodos:**

Manipulou-se o espaço negro entre os incisivos centrais superiores, para ambos os sexos, da seguinte forma: imagem controle, espaço negro de 1 mm, espaço negro de 2 mm e espaço negro de 3 mm. Participaram desse estudo noventa avaliadores, divididos em três grupos: 30 leigos, 30 cirurgiões-dentistas e 30 ortodontistas. Após a calibração visual de cada observador, foram projetadas oito fotografias no *software* OGAMA^®^ em conjunto com o *hardware* The Eye Tribe^®^. O OGAMA gerou informações do rastreamento do olhar de cada avaliador com relação ao tempo até a primeira fixação, mapa de calor, trajetória do olhar e tempo total de fixação para avaliar as áreas consideradas de interesse, de acordo com os avaliadores. Posteriormente, utilizou-se a EVA para avaliar as imagens a partir de um álbum, em uma escala de 0 a 10 pontos.

**Resultados:**

Os olhos e a boca foram as áreas com maior fixação pelos avaliadores, de acordo com os mapas de calor. Os resultados estatísticos não apresentaram diferença estatisticamente significativa entre os três grupos de avaliadores (*p*> 0,05), quanto ao tempo até a primeira fixação. Porém, em relação ao tempo de fixação na boca, observou-se diferença estatisticamente significativa (*p*< 0,05) na comparação dos três grupos.

**Conclusão:**

O espaço negro apresentou um efeito negativo na percepção estética da face. O aumento de fixação na boca correspondeu ao aumento do tamanho do espaço negro.

## INTRODUCTION

Orthodontic treatment is focused on enhancing the facial aesthetics, function, and general appearance. Importantly, even the smallest details can affect the smile attractiveness.

Both the smile and facial attractiveness constitute important aesthetic and social factors,^1^ because attractiveness increases the social interactions and develops the personality.^1^ ^,^ ^2^ The seek for facial perfection augments the necessity of studying aesthetic perceptions, and the use of digital programs helps to achieve more satisfactory results for patients.^3^ ^,^ ^4^ In health sciences, the construction of the values and meaning of the corporal aesthetic is receiving increased interest, influencing individual’s identity construction and self-perception.^5^

Knowledge of the patient’s psychological, anatomical, and functional needs can lead to better detection of changes or defects that may alter the aesthetic perceptions of the smile and identify the existing problems, in order to improve the aesthetic outcomes of orthodontic treatment and increase the quality of life of the patients.^5^ ^,^ ^6^

Aesthetic perception is related to educational, cultural, socioeconomical and emotional contexts. Importantly, studies have shown that perception differs between orthodontists, nonorthodontists (i.e., dentists), and laypeople.^7^

Eye-tracking has been used in visual perception investigations for a long time.^8^ The technique has been continuously refined since the introduction of the first eye-tracking machine.^9^

Black space is also known by the terms “black triangle” or “gap” and can be the result of: inclination of the maxillary central incisors in the mesial or distal direction; bone loss; triangular formation of the maxillary central incisors; or lesions associated with plaque, trauma, or tooth loss.^9-11^ The existence of these spaces can alter the smile aesthetic, although the degree of impact depends on the self-evaluation of the patient.^9^ ^,^ ^11^

A study demonstrated that in 98% of cases the interdental papilla is considered complete when the distance between the alveolar crest and the area of contact between the maxillary central incisors is equal to or less than 5 mm. With a distance of 6 mm, the papilla is considered complete in 56% of cases and this number decreases in the case of a 7-mm distance (27% of the cases).^12^

Previous studies with an objective of establishing a relation between the black space and smile attractiveness have confirmed that the black space has a negative repercussion on the dental aesthetic.^13^ In addition, young people are more capable of detecting the black space, ranking the smile as less attractive when greater black space is present.^7^

Eye-tracking is a trusted technique used to study aesthetic visual perception.^14^ Attractive judgment using a visual analogue scale (VAS) is also a simple and objective method for evaluating aesthetic perceptions and for helping to compare results between groups of raters.^15^

Therefore, the aim of the present study was to evaluate the effects of different sizes and magnitudes of black space between the maxillary central incisors in both sexes, with regard to aesthetic perception, by using an attractive assessment VAS and eye-tracking technique. 

## MATERIAL AND METHODS

This study was approved by the Committee of Human Ethics and Research of *Pontifícia Universidade Católica do Paraná* (# 2.235.302). Photos of individuals of both sexes were used, excluding those with characteristics that alter the visual attention such as beards, tattoos, exaggerated makeup or exotic hairstyles.

Facial and intraoral photographs were obtained using a Canon XT camera (Canon Inc., Tokyo, Japan), 50mm Sigma macro lens and Sigma flash. All photographs were obtained in a proper studio, with a white background.

High-resolution photographs were selected by three experienced orthodontists. The aspects of normality regarding symmetry, volume and color were observed. The images were edited in the Photoshop CS5^®^ software (Adobe Inc., San Jose, CA, USA) by an experienced professional.

**Figure 1:**
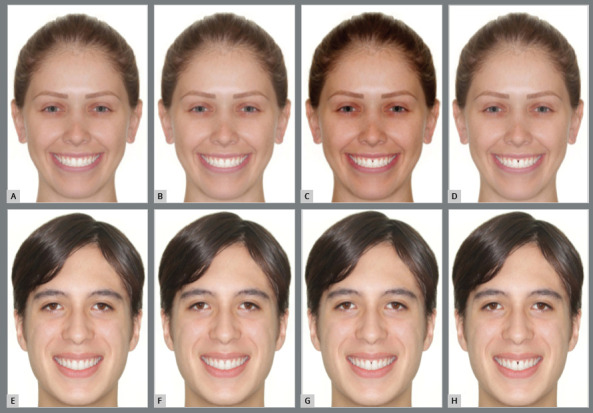
Black space on female patient: **A**) no black space; **B**) 1 mm, **C**) 2 mm, **D**) 3 mm. Black space on male patient: **E**) no black space; **F**) 1 mm, **G**) 2 mm, **H**) 3 mm.

Subsequently, the individuals’ actual smiles were excluded and another smile with better occlusion was inserted, to reduce the bias of the visual attention, because the original smiles were not symmetrical and the malocclusion could alter the visual attention and reduce the aesthetic perception. The aim of this study was to evaluate only the impact of the black spaces and its effect on the aesthetic perception.^14^ The black space was copied from a real photograph of a patient with black space, and then readapted to the study images.^13^ Black space between the central incisors was created, for both sexes, as follows:, Control, 1-mm black space, 2-mm black space and 3-mm black space (Fig 1).

The Ogama software (Freie Universität, Berlin, Germany) was used to perform eye-tracking, along with the The Eye Tribe tracking device. The Ogama software (OpenGazeAndMouseAnalyzer) was created and used to record and analyze eye movements based on multiple slide stimuli. This software provides qualitative (heatmap and scanpath) and quantitative analysis (total time of fixation in milliseconds and relative transition value that explains the scanpath) findings. All images were randomly included in the software in accordance with guidelines generated by the website www.randomizer.org, as follows: 2-mm black space, female; 3-mm black space, female; 1-mm black space, female; 2-mm black space, male; and 1-mm black space, male; control black space, male; control black space, female; and 3-mm black space, male. Each included image was adjusted to be visualized for three seconds by the raters and separated by a slide with white and green colors for one second, so that the last point of fixation of the previous image did not interfere with the first fixation point of the next one.

**Figure 2:**
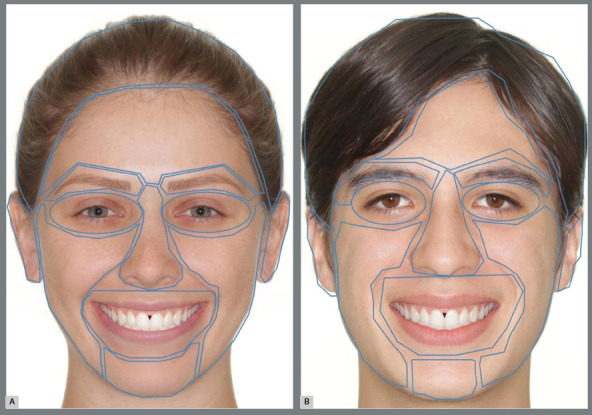
Delimited areas of interest of female and male faces.

Areas of interest (AOIs) in all images were also delimited by the Ogama software to obtain more accurate information about eye-tracking, as well as to make comparisons (Fig 2). The time to the first fixation (ms), fixation number, heatmap and scanpath of each evaluator were obtained for each AOI. All of the first fixations were eliminated as well as were the fixations with durations of less than 200 ms.

The distance used between the participant and The Eye Tribe hardware was 60 cm to 90 cm, as recommended by the manufacturer; and the height of the viewer’s chair was customized to capture the eyes in the center of a 23-inch monitor (Dell P2317H; Dell, Round Rock, TX, USA) positioned vertically. To begin the eye-tracking test, a standard eye movement calibration was performed and only excellent or good results were considered; the test was repeated when the result was poor or redo-commanded. When the test showed a poor or redo result twice, the participant was excluded from the study.

The raters were divided into three categories with 30 individuals each (layperson, nonorthodontist, and orthodontist). The following inclusion criteria were used: no previous neurological and/or vision impairment conditions; no recent use of drugs, alcohol, or medications that could interfere with cognitive abilities; Caucasian (to prevent bias due to race and/or nationality); and good eyesight (the use of glasses could interfere with the sensor). All volunteers were informed of the research conditions, risks and benefits, and signed a consent form.

After eye-tracking test, the same images were presented printed and organized in the same order, so that the raters could assess the attractiveness according to the VAS from zero to 10 points, with closer to zero points being less attractive, and closer to 10 points being more attractive.

## STATISTICAL ANALYSIS

The obtained results from eye-tracking and VAS were tabulated in the Microsoft Excel software (Microsoft Corp., Redmond, WA, USA) and analyzed in the Statistical Package for the Social Sciences software version 25 (IBM Corp., Armonk, NY, USA).

An analysis of variance (ANOVA) was used to demonstrate differences, comparing the mean values of the number of fixations and time to first fixation of the different groups of raters, as well as for VAS and each variable of the areas of interest (e.g., eyes, eyebrows, nose, mouth, forehead, hair, ears, and cheeks) with respect to the observer and the deviation (black space and diastema). For the independent sex variable, the Student’s *t*-test was used for independent samples. When the variables were continuous, to assess if there was an association between the two variables, the Pearson correlation coefficient was used.

When ANOVA indicated a statistically significant difference and the Levene homogeneity test indicated homogeneous variances, a two-way comparison was made, using Tukey’s multiple parametric comparison test for homogeneous variances. Otherwise the two-to-two comparison was made, using Games-Howell’s multiparametric comparison test for heterogeneous variances.

From the sample size of n = 30 or n > 30, considering the VAS variable for each category of the black space using SPSS software, the ANOVA method was applied to verify differences between observers and, in each dependent variable, was calculated the power of the test (observed power) for all cases in that there was a statistically significant difference. In none of the situations the power of the test was below 70%, and in most cases it was above 90%.

To correlate the mean values of the values assigned to the images with the VAS and the variables captured by the eye-tracking, the Pearson correlation test was used.

The final sample number of the survey among laypeople, nonorthodontists and orthodontists was 90, or 30 in each group. The age range of the raters was 18 to 46 years, with a mean age of 32 years. Regarding the sex of the participants, 52% (n = 47) were female and 48% were male (n = 43).

## RESULTS

The results of the eye-tracking showed that the mouth, right eye, and left eye were the most captured areas.

In relation to the time to first fixation and number of fixations of the mouth, dentists and orthodontists presented lower values than laypeople (*p*> 0.05) and there was no difference between nonorthodontists and orthodontists (*p*> 0.05) (Tables 1 and 2).

**Table 1:** Comparison of variations between all raters.

	Visual analogue scale	Time (ms) to first fixation (right eye)	Time (ms) to first fixation (mouth)	Number of fixations (right eye)	Number of fixations (mouth)
p value between groups	0.002	0.904	0.004	0.132	0.001

**Table 2:** Comparison between the three groups, by pairs.

	Visual analogue scale	Time (ms) to first fixation (right eye)	Time (ms) to first fixation (mouth)	Number of fixations (right eye)	Number of fixations (mouth)
Dentist x Orthodontist	0.316	0.915	0.904	0.109	0.065
Layperson x Dentist	0.128	0.915	0.007	0.441	0.441
Layperson x Orthodontist	0.001	1.000	0.015	0.627	0.001

In relation to attractive judgment based on the VAS, it was observed that the aesthetic perception decreased as the magnitude of the black space increased and that the highest notes were given for the images without any smile problems (Table 3).

**Table 3:** Mean and standard deviation of the evaluations, based on the visual analogue scale (VAS) between the different groups of raters.

VAS X GROUPS	Layperson	Nonorthodontist	Orthodontist	*p* **value**
	Mean (SD)			
1-mm black space female	6.1875 (2.10)	6.625 (2.01)	5.8 (1.88)	0.373
2-mm black space female	5.4688 (2.16)	4.9688 (2.65)	3.9 (2.07)	0.542
3-mm black space female	4.8125 (2.44)	4.375 (2.61)	3.8 (1.99)	0.695
Control female	8.125 (2.01)	8.4063 (1.47)	8.675 (1.54)	0.389
1-mm black space male	6.28 (2.53)^a^	5.75 (2.74)^b^	5.45 (1.97)^b^	0.001
2-mm black space male	4.4688 (2.46)	4.2188 (2.45)	3.025 (2.12)	0.116
3-mm black space male	3.5938 (2.35)	3.3125 (2.29)	2.175 (2.22)	0.407
Control male	6.5313 (2.79)^a^	7.5313 (2.69)^a^	8.75 (1.29)^b^	0.000

Different superscript letters indicate a statistically significant difference; statistically significant at P<0.05.

VAS = Visual Analogue Scale. SD = Standard Deviation.

A statistically significant difference (*p*< 0.05) was found when comparing the VAS outcomes between groups of raters. In the multicomparison of the values of the visual analogue scale, there was a statistically significant difference between the images of the female individuals without black space *versus* the images with black spaces of 1, 2, and 3 mm (*p*< 0.05).

**Figure 3:**
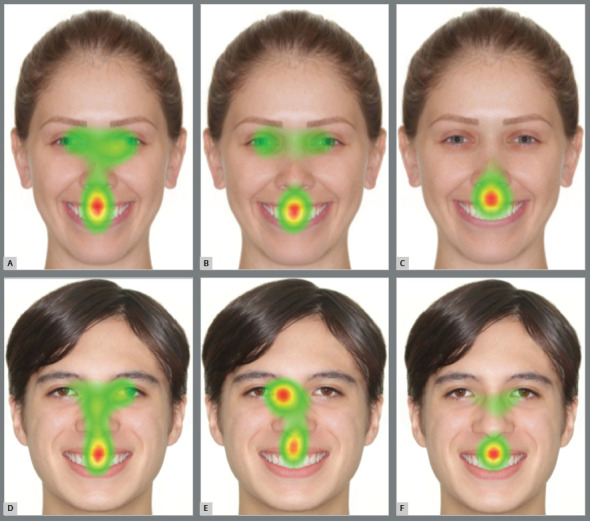
Heatmaps of the images of female patient with black space of 1mm: A) Laypeople, B) nonorthodontists and C) orthodontists. Heatmaps of the images of male patient with black space of 1mm: D) Laypeople; E) nonorthodontists and F) orthodontists.

**Figure 4:**
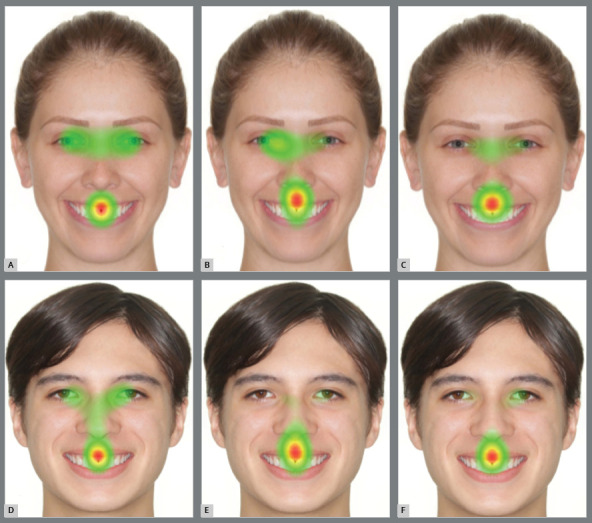
Heatmaps of the images of female patient with black space of 3mm: A) Laypeople, B) nonorthodontists and C) orthodontists. Heatmaps of the images of male patient with black space of 3mm: D) Laypeople; E) nonorthodontists and F) orthodontists.

Regarding the results of the heatmaps, it was shown that laypeople looked more to the eyes, in comparison to nonorthodontists and orthodontists, while the concentration of the gaze on the mouth increased as black spaces of 1, 2, and 3 mm appeared (Figs 3 and 4).

For nonorthodontists and orthodontists, the heatmaps demonstrated that the concentration on the mouth was greater than for laypeople, regardless of the magnification of black spaces in both sexes, mainly for orthodontists (Figs 3 and 4).

The scanpaths and the heatmaps revealed that the rater eye focus was correspondent to the size of the black space, especially for orthodontists. Upon evaluating the scanpaths of the control images, the eyes and the mouth were deemed to be the regions with greater focus.

## DISCUSSION

This study verified, by means of eye-tracking and visual analogue scale (VAS), the influence of black space on the general perception of face aesthetics evaluated by laypeople, nonorthodontists and orthodontists. It was verified that there is a negative effect of black space on general aesthetic perception of the face and smile. 

Other studies of aesthetic perception have compared these groups of individuals.^16^ ^,^ ^17^ The results confirmed that all groups evaluated negatively the images of 3-mm black space in both sexes and that the orthodontists were the most critical observers. However, the three rater groups also demonstrated a negative perception of black space of 1 mm and 2 mm.

The mouth or the lower third of the face was used in most prior studies of aesthetic perception. The study by Dindaroglu et al.^20^ used a cropped area of the face between the eyes and chin, without ears or hair. In the present study, images of the full face of both sexes were used, considering that, in everyday social relations, individuals have a general visual of the face in conjunction with the smile, not only an isolated region.

In this study, with the two techniques, it was possible to observe lower scores, referring to the images with larger black spaces and the raters concentrated on the mouth region. This can be explained by the fact that the existence of black space reduces the aesthetic perception of the face. However, no correlation was observed between the values of the VAS and the variables analyzed by eye-tracking.

The heatmaps of the eye-tracking showed that the eyes and mouth were the most interesting areas for all groups, showing an aesthetic relationship between the eyes and the mouth. However, the concentration toward the mouth was increased as black spaces were inserted in the photographs, suggesting the worsening of the aesthetics, confirmed by the evaluations through the VAS. Other studies that used eye-tracking also observed the same pattern of visualization between the eyes and mouth, and an increased concentration on the mouth region when black spaces were added to the smile.^14^ ^,^ ^21^

Besides that, it was also demonstrated that, in relation to time to first fixation in the mouth, there was a statistically significant difference between laypeople and nonorthodontists and between laypeople and orthodontists. As the area of concern for these professionals is the mouth, it is natural that they looked faster to the smile. However, other studies using eye-tracking have confirmed that time to first fixation may not be a very reliable measure.^14^ ^,^ ^22^ ^,^ ^23^

In the present study, orthodontists showed a higher attention to the mouth in the presence or absence of black space, regardless of rater sex, and were also more critical in the evaluation through VAS. These results are in agreement with other studies of perception, in which orthodontists perceived defects of a smaller magnitude when compared to other groups of raters.^11^ ^,^ ^24^ ^,^ ^25^ The present results can be expected then, since these professionals are trained to diagnose several types of occlusal abnormalities involving aesthetics and function.

A perfect smile has a positive repercussion on the dental aesthetics.^26^Comparing the three groups, it can be seen with the heatmaps and scanpaths that a black space of 3 mm shifts the concentration from the eyes to the mouth, neglecting the other areas of the face, in all three groups. This findings suggests the negative effect of black space on the smile for the perception of aesthetic attractiveness. The presence of black space of 1 mm and 2 mm also negatively influenced the aesthetic perception, when compared to the same image without a defect. Richards et al.^14^ observed that unattractive smiles changed the attention of the observer’s eyes on faces with different degrees of attractiveness; these results are in agreement with the present study, in which the perfect smile gained the highest scores among the three groups.

The results of this research correspond also with the research of Pithon et al.,^7^ who showed that the presence of black space between the maxillary central incisors was an important factor to decrease the aesthetic attractiveness of the smile, and revealed that black space decreased the overall aesthetic attractiveness of the face.

Eliminating black space should be included in the dental treatment plan, to augment the aesthetic perception and patient’s satisfaction. Nowadays, many dental aesthetic solutions can be applied depending on the case of each patient, as reported by Tanaka et al.^27^

A limitation of this study is that the test was applied only in one location, so the results reflect only the perceptions of one community, not a nationwide group. 

## CONCLUSION

Black space, in the magnitudes of 1, 2, and 3 mm, negatively affected the aesthetic perceptions of male and female faces in the evaluation of laypeople, nonorthodontists, and orthodontists.

Orthodontists showed greater attention on the mouth region in relation to the nonorthodontists and laypeople.

Analysis of the aesthetic perceptions among laypersons, nonorthodontists, and orthodontists, based on the VAS, found that orthodontists were the most critical.

The raters focused the eyes more than the mouth in both sexes in the control images. However for the images with 1mm of black space, the focus shifted to the mouth for the male patient. Separately, in images of 2 mm and 3 mm of black space, the concentration of the raters was on the mouth in both sexes.
